# Raynaud's Phenomena

**Published:** 1901-08-31

**Authors:** 


### RAYNAUD'S PHENOMENA.

MR. JONATHAN HUTCHINSON, senior, lecturing recently at the London Hospital, in referring to a case of a man, aged 62, suffering from Raynaud's disease, said:—"I relate this case in order to mention to you a new symptom which I quite recently observed in reference to these phenomena. It also shows the value of making a practice of always listening to what your patient has to tell you, else you may overlook very important details. My patient said to me, 'I have a red mark in my nails.' I looked at his nails, and I saw a red line exactly where the nail leaves its bed. This showed itself on all the nails, and was indeed very conspicuous. This redness could not be removed on pressure. It was quite clear he had a condition of thrombosis on the line of the capillaries at the very ends of all his nails. Now I am interested in the collection of portraits of nails, and I looked up two or three other photographs I had of cases with Raynaud's phenomena, and I saw my artist had accurately the pink fringe at the base of the nails. I had altogether overlooked this sign. I think we may add that to our symptoms of Raynaud's phenomena."

